# Interactive effects of maize straw incorporation and improved irrigation on soil physicochemical properties and microbial community structure in saline–alkaline soil

**DOI:** 10.3389/fmicb.2026.1752596

**Published:** 2026-02-04

**Authors:** Wenbo Chai, Hanyuan Xu, Chao Yuan, Shufen Li, Hongtao Li, Qing Zhu, Wei Ji, Ming Chi, Jun Wang, Chongxing Xin

**Affiliations:** Lianyungang Academy of Agricultural Sciences, Lianyungang, China

**Keywords:** enzyme activity, functional gene prediction, improved irrigation, maize straw incorporation, microbial community structure, saline–alkali soil, soil physicochemical properties

## Abstract

**Introduction:**

Saline–alkali soils severely limit agricultural Q7 productivity and ecological sustainability, particularly in coastal regions.This study evaluated the interactive effects of maize straw incorporation and improved irrigation on soil physicochemical properties, enzyme activities, and microbial community structure in saline–alkali soil through a field experiment.

**Methods:**

Soil organic matter, nitrogen forms, available nutrients, salinity, and electrical conductivity were determined, together with key enzyme activities (sucrase and polyphenol oxidase) and bacterial and fungal community composition. Microbial functional potential was further explored using functional gene prediction.

**Results:**

Compared with the control, the combined treatment significantly increased soil organic matter by 86.73% and available nutrients by 59.28%, while markedly reducing soil salinity and electrical conductivity by 53.56 and 47.12%, respectively. Soil enzyme activities responded differentially, with sucrase activity increasing by 109.23% and polyphenol oxidase activity decreasing by 29.35% compared with the control. Microbial community composition shifted substantially, characterized by a decline in halotolerant taxa (e.g., Flavobacteriaceae) and enrichment of carbon-cycling–associated bacteria (e.g., Cyanobiaceae), along with increased relative abundance of functional fungi such as Psathyrellaceae. Functional gene prediction revealed significant upregulation of carbohydrate metabolism pathways, including glycolysis and the tricarboxylic acid (TCA) cycle, indicating enhanced microbial metabolic capacity.

**Discussion:**

Overall, maize straw incorporation combined with improved irrigation effectively enhanced soil quality, reshaped microbial community structure, and strengthened microbial functional potential. These findings provide practical evidence that integrated straw–irrigation management can serve as a feasible and sustainable strategy for the remediation and productive utilization of saline–alkali soils.

## 1 Introduction

Saline-alkali land is a widely distributed land resource that poses a serious constraint on global agricultural sustainability. According to FAO and UNESCO estimates, saline-alkali soils cover approximately 954 million hectares worldwide, mainly in arid and semi-arid regions such as China, Australia, Russia, and the United States (Metternicht, 2003; Li et al., 2005; Yu et al., 2021). Soil salinization leads to vegetation degradation, reduced soil fertility, and substantial yield losses, thereby threatening food security (Bao et al., 2022; Ding et al., 2023; Ma et al., 2025). In China alone, more than 16 million hectares of land are affected by salinization, accounting for about 7.2% of the national arable land, with major distributions in the inland northwest, Huang–Huai–Hai Plain, northeastern lowlands, and coastal regions (Wang et al., 2017). Although engineering measures and agronomic practices such as straw incorporation and irrigation have been widely applied to ameliorate saline-alkali soils, their effects are often evaluated independently. In particular, how the interaction between straw return and irrigation regimes regulates soil microbial functional adaptation under saline-alkali stress remains poorly understood. Addressing this gap is critical for optimizing management strategies aimed at improving soil function and sustainable productivity in saline-alkali agroecosystems.

Among these, coastal saline-alkali lands are predominantly located along the approximately 18,000 km coastline from Liaoning to Guangxi, covering about 2.17 million hectares, of which nearly 95% are intertidal mudflats. Influenced by seawater intrusion, groundwater fluctuations, and poor drainage conditions, these soils are characterized by high salinity and alkalinity and low organic matter content, making saline-alkali land a dominant landscape type (Ren et al., 2012; Li et al., 2025). Coastal provinces such as Jiangsu, Liaoning, Hebei, and Shandong contain large and concentrated areas of saline-alkali land. Jiangsu Province alone has approximately 383,300 hectares of coastal saline-alkali mudflats, accounting for about one-fourth of China’s total coastal mudflat area and nearly one-tenth of its existing arable land (Yu et al., 2015). Despite the substantial potential for agricultural development, the high salinity and alkalinity of these soils pose major challenges to sustainable farmland utilization and ecological restoration.

To alleviate the agricultural and ecological constraints imposed by saline-alkali land, extensive efforts have been devoted to soil improvement technologies worldwide. Traditional remediation practices—including salt drainage, irrigation combined with fertilization, and topsoil replacement—have been shown to improve soil physicochemical properties to varying degrees (Murtaza et al., 2017; Zhao et al., 2021; Rezapour et al., 2022; Zhao Y. et al., 2023). However, these approaches are often associated with high economic costs, potential environmental risks, and limited long-term sustainability. In recent years, progress has been made in Jiangsu’s coastal saline-alkali land improvement through irrigation optimization, salinity regulation, soil structure enhancement, and the introduction of salt-tolerant crops. Nevertheless, key challenges remain, including low improvement efficiency and slow recovery of soil ecosystem functions (Shan et al., 2012; Yang, 2014; Wang G. et al., 2024). Therefore, the development of cost-effective and environmentally friendly remediation strategies remains a pressing need.

Maize is the second most widely cultivated crop worldwide, and maize straw represents one of the three largest agricultural residues globally, with considerable potential for resource utilization (Aghaei et al., 2022). Straw incorporation into soil has been widely reported to enhance soil fertility by increasing organic matter content, improving soil structure, and promoting nutrient availability (Liu et al., 2024; Zhao Y. et al., 2023, Wang G. et al., 2024). However, the effectiveness of maize straw utilization varies across regions, and its role in saline-alkali land remediation remains insufficiently explored. Previous studies have indicated that, under saline conditions, straw incorporation may be more effective than biochar in the short term for improving soil quality and crop productivity, partly by alleviating salt stress and reshaping microbial community structure (Cong et al., 2025; Song et al., 2025).

The combined application of maize straw incorporation and improved irrigation represents an integrated management strategy that couples organic resource recycling with hydrological regulation in saline–alkali soils. Straw decomposition supplies organic carbon and nutrients, while optimized irrigation regulates soil water–salt dynamics and alleviates salinity stress. We hypothesized that their synergistic application would simultaneously improve soil nutrient status, reduce salinity, and promote adaptive restructuring of soil microbial communities toward more functionally efficient assemblages.

To test this hypothesis, this study aimed to (i) quantify the combined effects of maize straw incorporation and improved irrigation on soil physicochemical properties and enzyme activities, (ii) characterize bacterial and fungal community responses across soil depths, and (iii) assess shifts in microbial functional potential using functional gene prediction. By integrating soil properties, enzyme activities, and microbial community dynamics, this study provides mechanistic insights into straw–irrigation management for the sustainable utilization and ecological restoration of coastal saline–alkali land.

## 2 Materials and methods

### 2.1 Study area overview

The field experiment was conducted at Wutuhe Farm, a coastal saline–alkali land in Jiangsu Province, China (34°26’ N, 119°38’ E). The region is characterized by a warm temperate marine monsoon climate with distinct seasons. Winters are cold and dry, whereas summers are hot and rainy, with peak temperature and precipitation occurring simultaneously. Spring and autumn show pronounced climatic variability. The annual sunshine duration is approximately 2,456.2 h, with a sunshine percentage of 55%. The mean annual temperature ranges from 13 to 15 °C, and the annual precipitation is 800–900 mm.

### 2.2 Experimental design

We designed a field experiment to evaluate the effects of maize straw incorporation combined with irrigation improvement on saline–alkali soil properties and microbial communities. Two treatments were established: (i) Control (CK): no straw incorporation with traditional local irrigation management, (ii) Straw + improved irrigation (SI): maize straw incorporation combined with irrigation optimization. Each treatment was arranged in a randomized block design with three replicates (plots were separated by buffer zones to avoid water and nutrient interference). For the SI treatment, naturally air-dried maize straw was crushed and incorporated into the soil at a rate of 3,000 kg ha^–1^ (≈200 kg mu^–1^). Straw was evenly mixed into the topsoil by plowing. Subsequently, irrigation was applied to maintain a surface water layer of approximately 10 cm, and the soil was soaked for 1 month before drainage. After drainage, soil moisture was maintained under field conditions for an additional 1 month to facilitate straw decomposition. The CK plots received no straw input and followed the same irrigation schedule traditionally used in the area.

Soil sampling was conducted before treatment implementation (YCK) and after the improvement period (YD). Samples were collected using a 4.5 cm diameter soil auger following a five-point sampling method. Two soil layers were sampled: 0–10 cm (a) and 10–20 cm (b). Collected samples were homogenized after removing stones and plant residues and passed through a 2 mm sieve. Each sample was divided into two subsamples: one stored in sterile tubes under liquid nitrogen for microbial sequencing, and the other used for physicochemical and enzyme analyses.

### 2.3 Determination of soil physicochemical properties and enzyme activities

Soil samples for physicochemical analyses were air-dried at room temperature in the shade, gently ground, and sieved through a 40-mesh screen. Soil organic matter was determined using the potassium dichromate oxidation method. Ammonium nitrogen (NH_4_^+^–N) and nitrate nitrogen (NO_3_^–^–N) were measured by continuous flow analysis. Available phosphorus and available potassium were determined using standard extraction methods. Soil pH was measured in a soil–water suspension (1:5, w/v) using a pH meter. Electrical conductivity (EC) and soil salinity were measured using a conductivity meter following aqueous extraction. Soil enzyme activities were determined using established colorimetric assays: polyphenol oxidase activity by pyrogallol oxidation, catalase activity by KMnO_4_ titration of residual H_2_O_2_, alkaline phosphatase activity using p-nitrophenyl phosphate as substrate, and sucrase activity based on glucose release quantified by 3,5-dinitrosalicylic acid (DNS) colorimetry (Li et al., 2019; Jia et al., 2020; Li et al., 2022; Ji et al., 2024).

### 2.4 DNA extraction and high-throughput sequencing

Total genomic DNA was extracted from soil samples using a commercial soil DNA extraction kit according to the manufacturer’s protocol. DNA quality and concentration were assessed using 1% agarose gel electrophoresis and spectrophotometry. Bacterial and fungal communities were analyzed by amplifying the 16S rRNA gene and ITS region, respectively. The bacterial primers were 16S_F (5’-AGAGTTTGATCCTGGCTCAG-3’) and 16S_R (5’-AAGGAGGTGATCCAGCCGCA-3’), while the fungal primers were ITS1_F (5’-TCCGTAGGTGAACCTGCGG-3’) and ITS2_R (5’-GCTGCGTTCTTCATCGATGC-3’). PCR amplification was performed using KAPA HiFi DNA Polymerase in an ABI GeneAmp^®^ 9700 thermal cycler, with three technical replicates per sample. Amplified products were pooled, verified by 2% agarose gel electrophoresis, purified using the AxyPrep DNA Gel Extraction Kit, and eluted with Tris–HCl buffer. For long-read sequencing, libraries were constructed following standard protocols for Illumina Platform.

### 2.5 Statistical analysis

OTU clustering was performed using QIIME and vsearch at 97% sequence similarity. Taxonomic assignment and community composition analyses were conducted using QIIME2. Phylogenetic trees at the genus level were constructed by sequence alignment with MAFFT and tree building using FastTree. Ternary plots were generated using the *grid* package in R after log_2_ transformation of relative abundance data. Differences among treatments were analyzed using STAMP software.

Functional annotation of bacterial communities was predicted using FAPROTAX. Data visualization was performed with Origin 9.1, and statistical significance among treatments was tested using SPSS, one-way analysis of variance (ANOVA) was first conducted to determine whether there were overall significant differences among groups; if significant differences existed (*p* < 0.05). Redundancy analysis (RDA) was conducted using Canoco 5.0 to assess relationships among soil physicochemical properties, enzyme activities, and microbial (bacterial and fungal) community structures; arrow length and angle in RDA biplots indicate the strength and direction of correlations, respectively.

## 3 Results

### 3.1 Effects of saline-alkali land improvement on soil physicochemical properties

**FIGURE 1:**
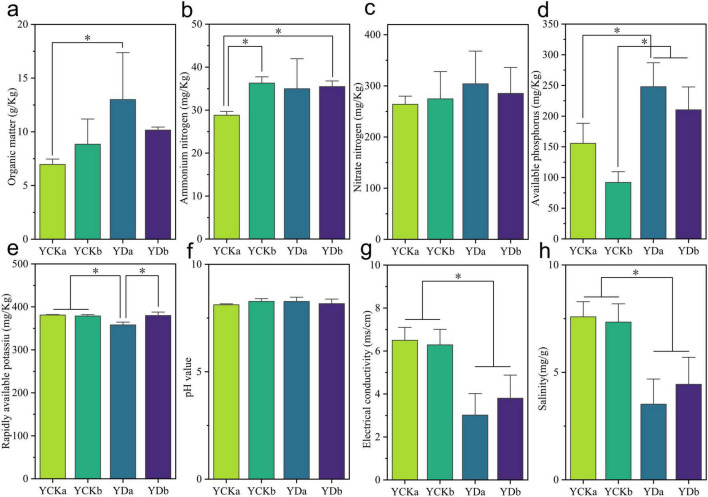
Effects of saline-alkali land improvement on soil physicochemical factors. **(a)** Organic matter content; **(b)** ammonium nitrogen content; **(c)** nitrate nitrogen content; **(d)** available phosphorus content; **(e)** available potassium content; **(f)** pH value; **(g)** electrical conductivity; **(h)** Salinity. Significance is indicated by * when *p* < 0.05.

Soil samples from two depths (0–10 cm and 10–20 cm) were analyzed before (YCK) and after (YD) saline–alkali land improvement to evaluate the effects of maize straw incorporation and improved irrigation on soil physicochemical properties, including organic matter, ammonium nitrogen, nitrate nitrogen, available phosphorus, available potassium, electrical conductivity (EC), pH, and salinity (Figure 1 and Supplementary Table S1). After improvement, soil organic matter content increased markedly, particularly in the surface layer (0–10 cm), rising from 6.96 ± 0.48g⋅kg^–1^ to 13.00 ± 3.78 g⋅kg^–1^—an increase of 86.73% (*p* < 0.05) (Figure 1a). In the deeper layer (10–20 cm), organic matter also increased by 14.87%, though the change was not statistically significant (*p* > 0.05). Both ammonium nitrogen and nitrate nitrogen showed increasing trends following improvement (Figures 1b,c). In the surface soil, ammonium nitrogen rose by 21.38% (from 28.82 ± 1.37 mg⋅kg^–1^ to 34.98 ± 6.05 mg⋅kg^–1^), and nitrate nitrogen by 15.15% (from 264.00 ± 16.00 mg⋅kg^–1^ to 304.00 ± 55.43 mg⋅kg^–1^). Similar but statistically insignificant increases were observed in the deeper soil layer (*p* > 0.05). Available phosphorus content increased significantly after improvement (Figure 1d), with increments of 59.28 and 128.35% in the surface and deeper layers, respectively (*p* < 0.05), showing a more pronounced enrichment at 10–20 cm. Conversely, available potassium exhibited a decreasing trend (Figure 1e), with a significant reduction in the surface soil (*p* < 0.05), while changes in the deeper layer were minor (*p* > 0.05). Soil pH remained relatively stable before and after improvement (Figure 1f), showing no significant variation at either depth (*p* > 0.05). However, EC and salinity decreased significantly after improvement (Figures 1g,h), particularly in the surface soil. Salinity in the 0–10 cm layer decreased by 53.56% (from 7.59 to 3.52 mg⋅g^–1^, *p* < 0.05), while in the 10–20 cm layer, it declined by 39.50% (from 7.34 mg⋅g^–1^ to 4.44 mg⋅g^–1^, *p* < 0.05).

### 3.2 Effects of saline-alkali land improvement on soil enzyme activities

**FIGURE 2:**
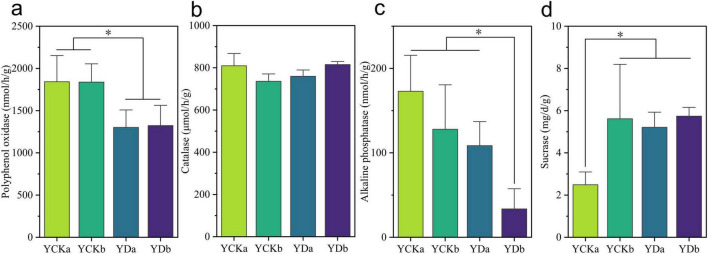
Effects of saline-alkali land improvement on soil enzyme activities. **(a)** Polyphenol oxidase; **(b)** catalase; **(c)** alkaline phosphatase; **(d)** sucrase. Significance is indicated by * when *p* < 0.05.

To evaluate the regulatory effects of maize straw incorporation and improved irrigation on soil biochemical processes, the activities of polyphenol oxidase, catalase, alkaline phosphatase, and sucrase were measured in two soil layers (0–10 cm and 10–20 cm) before (YCK) and after (YD) saline–alkali land improvement (Figure 2). Polyphenol oxidase activity decreased significantly after improvement (Figure 2a). In the surface soil (0–10 cm), activity declined from 1841.64 ± 289.38 nmol⋅h^–1^g⋅^–1^ to 1301.04 ± 179.49 nmol⋅h^–1^g⋅^–1^, representing a 29.35% reduction (*p* < 0.05). A similar trend was observed in the deeper layer (10–20 cm), where activity decreased by 28.04% (from 1838.04 ± 290.17 nmol⋅h^–1^g⋅^–1^ to 1322.67 ± 305.63 nmol⋅h^–1^g⋅^–1^, *p* < 0.05). Catalase activity showed opposite but statistically insignificant trends across soil layers (Figure 2b). It slightly decreased in the surface soil (0–10 cm) but increased marginally in the deeper layer (10–20 cm) (*p* > 0.05). Alkaline phosphatase activity decreased significantly following improvement (Figure 2c). The decline was particularly evident in the deeper soil, where activity dropped from 127.91 ± 46.54 mg⋅d^–1^g⋅^–1^ to 33.52 ± 21.36 mg⋅d^–1^g⋅^–1^—a 73.80% reduction (*p* < 0.05). Although a decrease was also observed in the surface soil, it was not statistically significant (*p* > 0.05). In contrast, sucrase activity exhibited a significant increase after improvement (Figure 2d). In the surface soil, activity rose from 2.49 ± 0.77 mg⋅d^–1^g⋅^–1^ to 5.21 ± 0.96 mg⋅d^–1^g⋅^–1^, an increase of 109.23% (*p* < 0.05). A slight, non-significant increase was also detected in the deeper soil (*p* > 0.05).

### 3.3 Distribution characteristics of soil microbial OTUs

**FIGURE 3:**
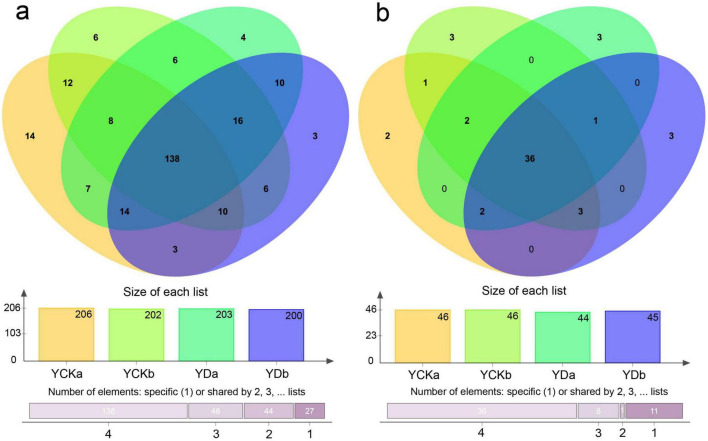
Venn diagrams illustrating the overlap of soil microbial communities among treatments. **(a)** Bacterial communities; **(b**) Fungal communities.

High-throughput sequencing technology was used to analyze soil microbial communities (bacteria and fungi) in different soil layers (0–10 cm and 10–20 cm) before (YCK) and after (YD) saline-alkali land improvement. Clustering at 97% sequence similarity identified 811 bacterial OTUs and 101 fungal OTUs (Figure 3). Among bacterial OTUs, shared OTUs accounted for 17.02% of the total OTUs, while unique OTUs in the YCKa, YCKb, YDa, and YDb samples comprised 6.80, 2.97, 1.97, and 1.50% of their respective total OTUs (Figure 3a). For fungal OTUs, shared OTUs constituted 35.64% of the total OTUs. The proportions of unique OTUs in the YCKa, YCKb, YDa, and YDb samples were 4.35, 6.52, 6.82, and 6.67%, respectively (Figure 3b).

### 3.4 Analysis of soil microbial community composition

**FIGURE 4:**
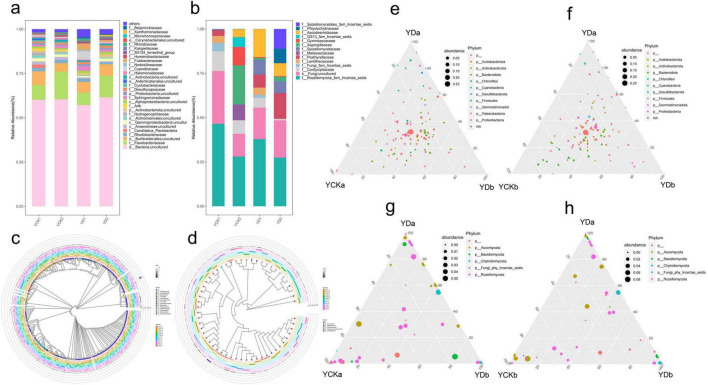
Microbial community classification analysis. **(a,b)** Microbial abundance bar chart; **(c,d)** OTU phylogenetic tree; **(e–h)** Taxonomic distribution plots.

The taxonomic composition of soil bacterial and fungal communities was analyzed at the family level in the 0–10 cm and 10–20 cm soil layers before (YCK) and after (YD) saline–alkali land improvement (Figure 4).

Improvement measures markedly altered the composition and distribution of soil bacteria (Figure 4a). The relative abundance of Flavobacteriaceae declined significantly, particularly in the deeper layer, indicating reduced dominance of halotolerant taxa. In contrast, Cyanobiaceae increased substantially, especially in the surface soil, suggesting enhanced adaptability under improved conditions. Rhodobacteraceae showed a slight decrease overall, but its distribution shifted toward the surface layer after improvement. Sphingomonadaceae abundance increased significantly, with a more uniform distribution between layers. The low-abundance Fusibacteraceae exhibited a pronounced increase, particularly in surface soil, while Cyclobacteriaceae decreased consistently across layers. Halomonadaceae remained relatively stable, showing little change before and after improvement. Vertical comparisons indicated that Flavobacteriaceae was consistently more abundant in the deeper layer, whereas Cyanobiaceae predominated in the surface soil, and this pattern became more distinct after improvement. The enrichment of Fusibacteraceae suggested a strong positive response to the integrated management measures, while the decline of Cyclobacteriaceae reflected the reduced salinity stress.

Fungal community structure also changed notably following improvement (Figure 4b). The abundance of Aspergillaceae decreased significantly, whereas Psathyrellaceae, Ascodesmidaceae, and Spizellomycetaceae increased, indicating that these taxa adapted well to the improved soil environment. In contrast, Gymnoascaceae, Cordycipitaceae, and Malasseziaceae markedly declined or nearly disappeared, suggesting poor tolerance to the reduced salinity and altered nutrient conditions. Lentitheciaceae exhibited a contrasting pattern, increasing slightly in the surface soil but decreasing significantly in the deeper layer. Phlyctochytriaceae showed the opposite trend, being enriched mainly in the deeper layer after improvement.

Across all samples, the dominant bacterial phyla included Proteobacteria, Bacteroidota, Firmicutes, Chloroflexi, Acidobacteriota, and Cyanobacteria (Figures 4c,e). These phyla encompassed multiple classes such as *Alphaproteobacteria*, *Bacteroidia*, *Clostridia*, and *Cyanobacteriia*. Representative genera included *Vicinamibacter*, *Salegentibacter*, *Gramella*, *Yoonia*, and *Fusibacter*. Some OTUs were identified at the species level, such as *Pontibacter salisaro*, *Gramella oceani*, and *Salinimicrobium sediminis*, reflecting the high microbial diversity of the studied soils.

Fungal communities were dominated by Rozellomycota, followed by Ascomycota, Basidiomycota, and Chytridiomycota (Figures 4d,f). Within Ascomycota, genera such as *Leucothecium*, *Halobyssothecium*, *Aspergillus*, and *Cordyceps* were identified, while *Malassezia* and *Coprinellus* represented the main Basidiomycota members. Although many OTUs within Rozellomycota were classified as *Rozellomycota_sp* (Incertae sedis), this group dominated the overall fungal assemblage, indicating its ecological prevalence in saline–alkali environments.

Overall, soil improvement significantly reshaped microbial community composition, reducing halotolerant and stress-adapted taxa while enriching carbon- and nutrient-cycling groups, thereby promoting a more functionally diverse and ecologically balanced microbial community.

Taken together, these results demonstrate that the saline–alkali land improvement treatment fundamentally restructured soil microbial communities across soil depths, shifting them from salinity-tolerant assemblages toward functionally diverse taxa associated with carbon turnover and nutrient cycling.

### 3.5 Weighted UniFrac distance analysis

**FIGURE 5:**
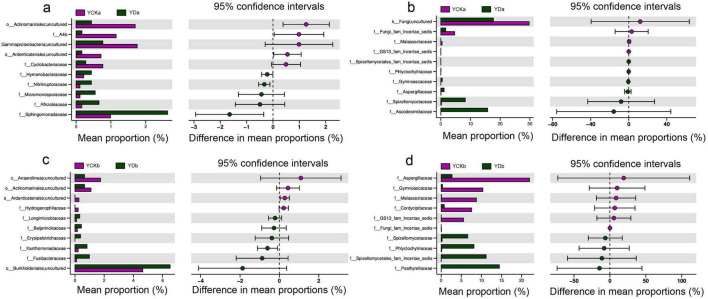
Weighted UniFrac-based β-diversity analysis of soil bacterial and fungal communities among treatments. **(a,b)** Bacterial communities. **(c,d)** Fungal communities. **(a,c)** Heatmaps of pairwise community dissimilarities calculated using the weighted UniFrac distance. **(b,d)** Bar plots summarizing between-group β-diversity differences.

The Weighted UniFrac distance analysis revealed clear differences in the beta diversity of microbial communities across treatments and soil depths (Figure 5). For bacteria (Figures 5a,b), the YCKa group (0–10 cm before improvement) exhibited the smallest pairwise distances (0.06–0.10), indicating high community similarity. In contrast, the YDa group (0–10 cm after improvement) showed the largest distances (up to 0.29), suggesting substantial heterogeneity following saline-alkali land improvement. The YDb group (10–20 cm after improvement) displayed moderate distances (0.10–0.15), reflecting relatively stable community composition in deeper soil. Overall, bacterial communities in the improved surface soil (YDa) exhibited significantly higher Weighted UniFrac distances than other groups (*p* < 0.05), indicating that improvement measures markedly altered surface bacterial community structure. For fungi (Figures 5c,d), larger pairwise distances were generally observed, indicating greater variability than in bacterial communities. The YCKb and YDa groups showed the highest intra-group distances (up to 0.76 and 0.68, respectively), reflecting strong internal heterogeneity. In contrast, the YDb group exhibited relatively small distances (0.41–0.52), suggesting a more stable fungal structure in deeper soil after improvement. Although the Weighted UniFrac distance of surface fungi (YDa) increased after improvement, the change was not statistically significant (*p* > 0.05), implying that fungal communities were less sensitive to improvement measures compared with bacteria.

### 3.6 Statistical test analysis of microbial families

**FIGURE 6:**
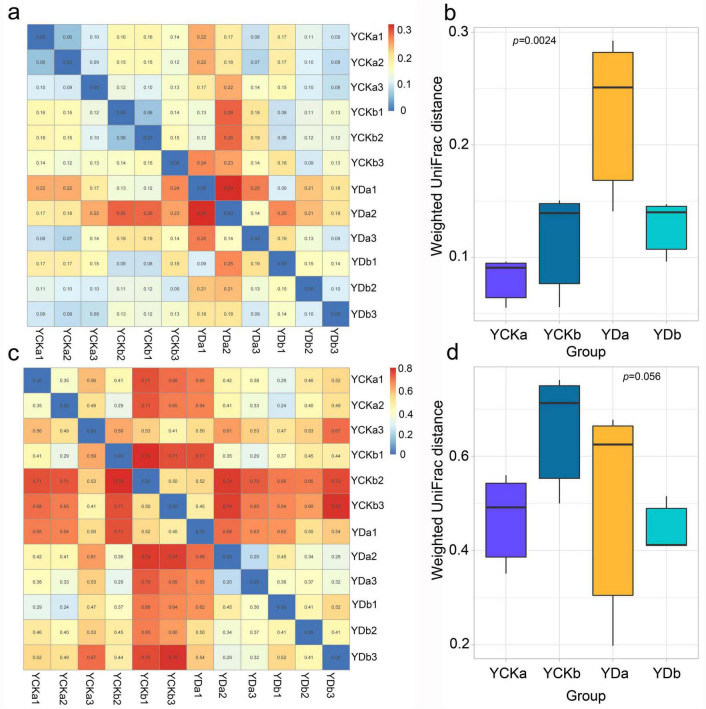
Differential abundance analysis of soil microbial communities between treatments across soil depths. **(a,c)** Bacterial communities. **(b,d)** Fungal communities. Panels **(a,b)** represent samples from the 0 to 10 cm soil layer, while panels **(c,d)** represent samples from the 10 to 20 cm soil layer.

Statistical comparisons of microbial communities before (YCK) and after (YD) saline-alkali land improvement revealed pronounced changes in community structure across soil depths (Figure 6). In the bacterial community, surface soil (0–10 cm) showed significant increases in the relative abundances of *Sphingomonadaceae*, *Rhizobiaceae*, *Micromonosporaceae*, and *Nitriliruptoraceae* after improvement, whereas *Cyclobacteriaceae*, *Ardenticatenales*, and *Gammaproteobacteria* declined (Figure 6a). In the deeper soil (10–20 cm), *Burkholderiales*, *Fusibacteraceae*, *Xanthomonadaceae*, *Erysipelotrichaceae*, *Beijerinckiaceae*, and *Longimicrobiaceae* increased significantly, while *Anaerolineae*, *Actinomarinales*, and *Hydrogenophilaceae* decreased (Figure 6c). For fungi, improvement promoted the growth of *Ascodesmidaceae*, *Spizellomycetaceae*, *Aspergillaceae*, and *Gymnoascaceae* in the surface soil (Figure 6b), while *Malasseziaceae* declined markedly. In deeper soil, *Psathyrellaceae*, *Phlyctochytriaceae*, and *Spizellomycetaceae* increased, whereas *Aspergillaceae*, *Gymnoascaceae*, *Malasseziaceae*, and *Cordycipitaceae* decreased (Figure 6d). Overall, these results demonstrate that saline-alkali land improvement significantly reshaped microbial community composition, with distinct bacterial and fungal responses across soil depths—reflecting the stratified ecological effects of straw incorporation and irrigation improvement.

### 3.7 Regulatory effects of saline-alkali soil improvement on soil microbial functional communities

**FIGURE 7:**
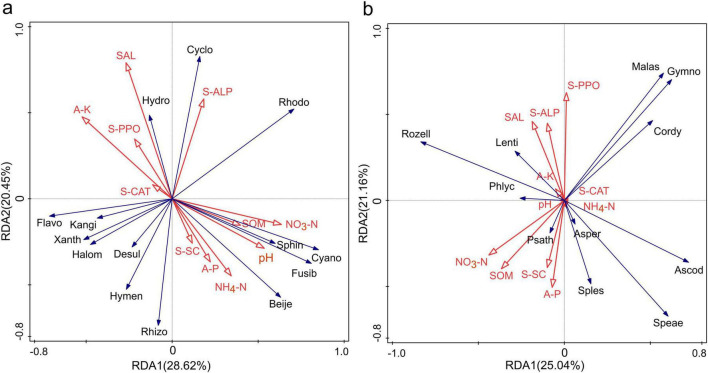
Functional abundance profiles of microbial communities. **(a)** Bacteria; **(b)** Fungi.

Functional prediction of bacterial communities (Figure 7a) revealed that saline-alkali soil improvement markedly reshaped microbial metabolic potential across soil depths (0–10 cm and 10–20 cm). The expression of genes involved in carbohydrate, lipid, amino acid, energy, nucleotide, and secondary metabolite metabolism was significantly upregulated after improvement. In carbohydrate metabolism, key pathways such as glycolysis/gluconeogenesis, the TCA cycle, and pentose phosphate and fructose–mannose metabolism were enhanced, suggesting improved microbial energy acquisition and carbon turnover. In lipid metabolism, genes related to fatty acid biosynthesis and ketone body metabolism increased notably, indicating enhanced lipid utilization capacity. Amino acid metabolism showed the most prominent changes, with strong upregulation of genes involved in valine, leucine, and isoleucine degradation, glycine–serine–threonine metabolism, and lysine biosynthesis, implying improved nitrogen cycling efficiency. Furthermore, oxidative phosphorylation and photosynthesis-related genes in energy metabolism, as well as purine and pyrimidine metabolism in nucleotide pathways, were significantly elevated. Genes involved in penicillin and cephalosporin biosynthesis also increased, suggesting enhanced secondary metabolic potential and microbial competitiveness under improved soil conditions. Functional analysis of fungal communities (Figure 7b) showed pronounced shifts in trophic guild composition following improvement. The Animal Pathogen–Clavicipitaceous Endophyte–Fungal Parasite and Animal Pathogen–Undefined Saprotroph groups, dominant in untreated soils, declined sharply after improvement, indicating suppression of pathogenic and parasitic fungi. Conversely, Dung Saprotroph–Plant Saprotroph–Wood Saprotroph and Dung Saprotroph–Soil Saprotroph groups increased markedly—particularly in deeper soils—reflecting enhanced saprotrophic activity and organic matter decomposition. Meanwhile, the Plant Pathogen–Undefined Parasite–Undefined Saprotroph group decreased substantially across both depths, suggesting a reduction in potential plant pathogens. Overall, saline-alkali land improvement enhanced the metabolic versatility and saprotrophic functions of soil microorganisms while reducing pathogenic and parasitic groups, thereby promoting a healthier and more functionally active microbial ecosystem.

## 4 Discussion

### 4.1 Effects of saline-alkali land improvement on soil physicochemical properties and enzyme activities

The present study demonstrates that the combined application of straw incorporation and irrigation improvement substantially enhanced soil physicochemical properties and enzymatic activities in saline-alkali land. A pronounced increase in soil organic matter (SOM) was observed, particularly in the surface layer (0–10 cm), where SOM increased by 86.73% (*p* < 0.05). This magnitude of SOM enrichment is comparable to or higher than that reported in previous studies on straw-return practices in saline or degraded soils, suggesting that the synergistic regulation of organic input and water–salt balance may amplify carbon accumulation efficiency (Chen et al., 2025). This enhancement indicates that straw incorporation effectively introduced labile organic carbon, stimulating microbial activity and accelerating organic matter turnover. In contrast, the modest 14.87% increase in deeper soil (10–20 cm) suggests limited downward migration of decomposition products or slower accumulation processes, a pattern consistent with depth-dependent carbon stabilization reported in amended saline soils. The concurrent rise in ammonium and nitrate nitrogen contents supports the notion that improvement measures promoted nitrogen mineralization and accumulation (Yu et al., 2024). Similar increases in inorganic nitrogen pools following straw incorporation have been widely documented and are often attributed to enhanced microbial decomposition and improved aeration conditions, particularly under optimized irrigation regimes. The enhanced nitrogen availability likely stemmed from straw-derived nitrogen release and improved soil aeration under irrigation. The significant increase in available phosphorus (A-P), particularly in the 10–20 cm layer (+128.35%, *p* < 0.05), may be attributed to vertical phosphorus migration through irrigation (Benelli et al., 2024) and the mobilization of insoluble phosphorus by organic acids released during straw decomposition (Yang et al., 2019; Zhao et al., 2024). Conversely, the decline in available potassium (A-K), especially in surface soil, may result from rapid potassium leaching after straw decomposition, a phenomenon frequently reported in straw-amended soils with intensified water fluxes. The marked reductions in soil salinity (-53.56%) and electrical conductivity (-47.12%) further confirm that irrigation effectively leached salts and ameliorated saline stress, improving the overall soil environment. Comparable declines in salinity following irrigation optimization have been reported in coastal and inland saline-alkali soils, reinforcing the effectiveness of hydrological regulation as a key driver of soil amelioration.

Enzyme activity responses revealed the biological regulation associated with these physicochemical changes. Polyphenol oxidase (S-PPO) activity declined significantly (-29.35% in 0–10 cm, *p* < 0.05), likely reflecting the reduced proportion of recalcitrant SOM substrates following the enrichment of labile organic matter (Sun et al., 2017). This response aligns with previous observations that PPO activity tends to decrease when easily decomposable carbon becomes dominant, indicating a shift toward faster carbon cycling pathways. Similarly, alkaline phosphatase (S-ALP) activity decreased markedly, particularly in the deeper soil (-73.80%, *p* < 0.05), which can be attributed to phosphorus enrichment that suppresses phosphatase synthesis via negative feedback (Allison and Vitousek, 2005; Zheng et al., 2024). In contrast, sucrase (S-SC) activity increased significantly (+109.23% in surface soil, *p* < 0.05), reflecting elevated carbon availability and enhanced microbial metabolic capacity (Wu et al., 2023).

Overall, these findings highlight the synergistic mechanisms underlying soil improvement: straw decomposition enriches SOM and nutrient pools, providing substrates and energy for microbial metabolism, while irrigation alleviates salt stress and improves nutrient mobility. The consistency of these responses with existing literature supports the robustness of the proposed mechanisms, suggesting that integrated straw–irrigation management is an effective approach for improving saline-alkali soil functioning.

### 4.2 Adaptive restructuring of microbial community under saline-alkali land improvement

The improvement of saline–alkali land markedly reshapes soil microbial diversity, with bacteria and fungi exhibiting distinct adaptive responses. Following the combined implementation of straw incorporation and irrigation optimization, bacterial operational taxonomic units (OTUs) increased significantly, especially in surface soil (0–10 cm). This enhancement can be attributed to the alleviation of salt stress and the restoration of a balanced soil water–salt environment. Similar increases in bacterial richness under reduced salinity conditions have been widely reported, indicating that salinity is a primary constraint on bacterial community expansion in saline-alkali soils. In contrast, bacterial richness and diversity decreased with soil depth, consistent with previous findings (Guo et al., 2022; Jiang et al., 2024). Fungal diversity, however, showed a different trend, with a notable increase in the 10–20 cm layer, likely reflecting the slower ecological responses and life-history strategies of fungi. This depth-dependent fungal response has also been observed in straw-amended soils, where fungi play a key role in the decomposition of more complex organic substrates. The reduction in halotolerant and alkaliphilic bacterial groups further confirms the stabilization of soil physicochemical conditions, favoring the establishment of non-halophilic taxa. Enhanced microbial diversity after improvement also implies an expansion of ecological niches, closely associated with elevated soil organic matter, optimized C/N ratios, and increased nutrient availability (Ma et al., 2024). These patterns collectively suggest that environmental filtering driven by salinity stress was weakened, allowing resource availability to become a dominant driver of community assembly.

Community compositional shifts further highlight the adaptive restructuring mechanisms of soil microbes. The relative abundance of halotolerant taxa such as Flavobacteriaceae and Rhodobacteraceae decreased following improvement, reflecting the loss of their competitive advantage as salinity declined. In contrast, Cyanobiaceae and Sphingomonadaceae increased markedly. These taxa are commonly associated with carbon transformation processes and nutrient cycling; however, their increased abundance in this study should be interpreted as an indicator of enhanced metabolic potential rather than direct evidence of elevated functional activity (Lee et al., 2018; Shan et al., 2024). Although Cyanobiaceae are generally associated with photosynthetic or mixotrophic metabolism, whether and to what extent these pathways contribute to carbon input in subsurface soils under improved saline conditions remains unclear, as this study did not directly assess their functional activity. For fungal communities, the enrichment of functional taxa such as Ascodesmidaceae and Psathyrellaceae suggests enhanced organic matter decomposition and nutrient cycling capacity (Baldrian, 2017; Wang et al., 2019). Nevertheless, these functional interpretations are based on taxonomic affiliations and should be regarded as putative, as direct measurements of enzyme production or substrate utilization were not conducted.

Overall, the observed microbial community shifts and diversity patterns, together with their close associations with improved soil physicochemical properties, indicate a coordinated microbial response to saline–alkali land improvement. However, the mechanisms underlying the enrichment of specific taxa, particularly Cyanobiaceae, remain largely inferential in this study and should be interpreted with caution. We hypothesize that the increase in Cyanobiaceae may be driven by (i) reduced osmotic stress and improved soil moisture facilitating photoautotrophic or mixotrophic growth in surface soil, (ii) enhanced carbon availability from straw-derived substrates supporting heterotrophic or facultative metabolic pathways, and/or (iii) altered redox and nutrient conditions favoring taxa with metabolic flexibility. These hypotheses are testable through future studies combining metagenomic or metatranscriptomic analyses with measurements of functional gene expression (e.g., carbon fixation, nitrogen metabolism), enzyme activities, and stable isotope tracing, which would allow direct verification of microbial functional responses and interaction mechanisms under saline–alkali land improvement.

### 4.3 Microbial functional reconstruction driven by soil improvement

It should be noted that functional predictions in this study were inferred based on taxonomic annotation using database-driven approaches. Such predictions provide valuable insights into potential metabolic functions but do not directly reflect actual gene expression or enzymatic activity. Therefore, the functional profiles should be interpreted with caution, and future studies integrating metagenomic or metatranscriptomic approaches are needed to further validate microbial functional responses.

Soil microbial functional analysis revealed that saline–alkali land improvement induced substantial changes in the expression of key functional genes across soil depths (0–10 cm and 10–20 cm). The combined application of straw return and irrigation significantly altered microbial metabolic functions, particularly in carbohydrate, lipid, amino acid, energy, nucleotide, and secondary metabolite pathways, indicating a fundamental restructuring of the microbial functional potential.

In carbohydrate metabolism, genes involved in glycolysis/gluconeogenesis and the tricarboxylic acid (TCA) cycle were markedly upregulated, suggesting more efficient decomposition and utilization of humic substances and enhanced microbial energy production (Nardi et al., 2007). The upregulation of genes related to the pentose phosphate and fructose/mannose pathways further indicated intensified carbohydrate metabolism and redistribution of carbon flux, reinforcing carbon flow and energy conversion in the soil ecosystem (Treseder, 2008). Lipid metabolism also showed significant upregulation of genes associated with fatty acid biosynthesis and ketone body synthesis/degradation. These changes imply enhanced membrane lipid dynamics, contributing to improved cell membrane stability and increased microbial tolerance to saline–alkali stress (Allison and Martiny, 2008). Amino acid metabolism pathways exhibited particularly strong responses, with genes related to valine, leucine, and isoleucine degradation, glycine, serine, and threonine metabolism, and lysine biosynthesis all upregulated. This indicates enhanced microbial nitrogen assimilation and amino acid synthesis efficiency, promoting nitrogen cycling and soil fertility while supporting stable nutrient availability for plant growth (Philippot et al., 2013).

In energy metabolism, the upregulation of oxidative phosphorylation and photosynthesis-related genes reflected improved microbial capacity for energy generation and storage, thereby increasing microbial activity and adaptability in the post-improvement environment. Similarly, in nucleotide metabolism, elevated expression of purine and pyrimidine metabolism genes suggested enhanced nucleotide synthesis and turnover, which facilitate cell proliferation and genomic stability, promoting the formation of a more resilient microbial community (Baldrian, 2017). Secondary metabolite metabolism was also significantly affected. The upregulation of genes involved in penicillin and cephalosporin biosynthesis indicates a strengthened microbial ability to produce antimicrobial compounds. This may enhance biological stress resistance and indirectly improve soil health and plant productivity by suppressing pathogenic microorganisms.

Overall, the integration of straw return and irrigation stimulated beneficial metabolic pathways—including carbon, nitrogen, and energy metabolism—while potentially suppressing harmful microbial functions. These functional reconstructions enhance nutrient cycling, organic matter turnover, and pathogen suppression, thereby improving the ecological sustainability of saline–alkali soils. The results provide a scientific basis and practical framework for large-scale ecological restoration and sustainable management of saline–alkali land.

### 4.4 Relationships among soil physicochemical factors, enzyme activities, and soil microbial communities

**FIGURE 8:**
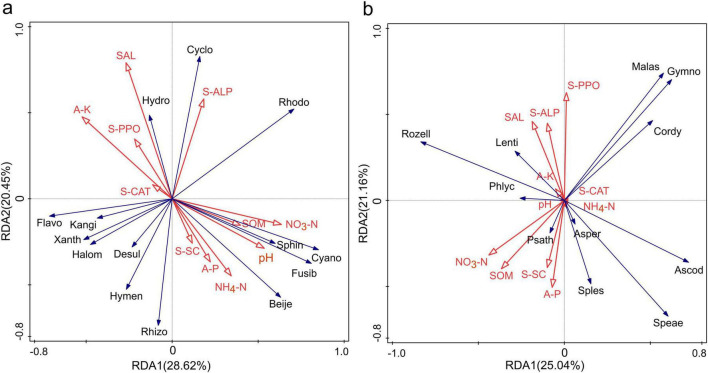
Redundancy analysis (RDA) of soil microbial communities in relation to soil physicochemical properties and enzyme activities. **(a)** Bacteria. **(b)** Fungi. SOM, soil organic matter; NH_4_^+^-N, ammonium nitrogen; NO_3_^–^-N, nitrate nitrogen; A-P, available phosphorus; A-K, available potassium; EC, electrical conductivity; pH, potential of hydrogen; SAL, soil salinity; S-PPO, soil polyphenol oxidase; S-CAT, soil catalase; S-SC, soil sucrase; S-ALP, soil alkaline phosphatase; Flavo, *Flavobacteriaceae*; Rhodo, *Rhodobacteraceae*; Sphin, *Sphingomonadaceae*; Cyano, *Cyanobiaceae*; Fusib, *Fusibacteraceae*; Desul, *Desulfocapsaceae*; Halom, *Halomonadaceae*; Cyclo, *Cyclobacteriaceae*; Hydro, *Hydrogenophilaceae*; Hymen, *Hymenobacteraceae*; Rhizo, *Rhizobiaceae*; Beije, *Beijerinckiaceae*; Kangi, *Kangiellaceae*; Xanth, *Xanthomonadaceae*; Rozel, *Rozellomycota*; Ascod, *Ascodesmidaceae*; Asper, *Aspergillaceae*; Cordy, *Cordycipitaceae*; Psath, *Psathyrellaceae*; Speae, *Spizellomycetaceae*; Sples, *Spizellomycetales*; Gymno, *Gymnoascaceae*; Malas, *Malasseziaceae*; Phlyc, *Phlyctochytriaceae*; Lenti, *Lentitheciaceae*.

For bacteria (Figure 8a), RDA1 and RDA2 explained 28.62 and 20.45% of the total variation, respectively, accounting for 49.07% overall. Soil Organic Matter (SOM), Ammonium Nitrogen (NH_4_^+^-N), Nitrate Nitrogen (NO_3_^–^-N), Available Phosphorus (A-P), Available Potassium (A-K), pH, Soil Salinity (SS), and enzyme activities (polyphenol oxidase, catalase, sucrase, and alkaline phosphatase) were major drivers of bacterial community distribution. Different bacterial families exhibited distinct ecological preferences. *Rhodobacteraceae* showed strong positive correlations with SOM, NH_4_^+^-N, NO_3_^–^-N, pH, and alkaline phosphatase, reflecting their preference for nutrient-rich and alkaline environments (Simon et al., 2017). *Cyclobacteriaceae* and *Hydrogenophilaceae* were positively associated with soil salinity and oxidative enzyme activities, indicating tolerance to saline and redox conditions. *Flavobacteriaceae* correlated with catalase, A-K, and polyphenol oxidase, suggesting sensitivity to oxidoreductase activity and salinity. *Kangiellaceae*, *Xanthomonadaceae*, and *Halomonadaceae* were primarily associated with catalase and A-K, implying adaptation to potassium-rich and oxidative environments. In contrast, *Desulfocapsaceae* and *Hymenobacteraceae* were positively related to sucrase, A-P, and NH_4_^+^-N, suggesting active carbon metabolism under high nutrient availability. *Rhizobiaceae*, *Beijerinckiaceae*, *Fusibacteraceae*, *Sphingomonadaceae*, and *Cyanobiaceae* also showed strong positive associations with SOM, NO_3_^–^-N, NH_4_^+^-N, A-P, and pH, reflecting their adaptive advantage in nitrogen- and phosphorus-enriched environments.

For fungi (Figure 8b), RDA1 and RDA2 explained 25.04 and 21.16% of the total variation, accounting for 46.20% overall. Fungal community composition was mainly influenced by soil polyphenol oxidase, alkaline phosphatase, salinity, nitrate nitrogen, SOM, sucrase, and available phosphorus. *Cordycipitaceae*, *Gymnoascaceae*, and *Malasseziaceae* were positively correlated with oxidase activities, alkaline phosphatase, and salinity, suggesting adaptation to oxidative and alkaline conditions. *Lentitheciaceae* also correlated with nitrate nitrogen, indicating an ecological preference for saline and nitrogen-rich environments. *Rozellomycota* exhibited positive associations with oxidase activities, SOM, salinity, and nitrogen, implying dominance in organic-rich, moderately saline conditions. *Phlyctochytriaceae* showed strong positive correlations with sucrase, A-P, SOM, salinity, and nitrate nitrogen, indicating dependence on active carbon, nitrogen, and phosphorus cycling. *Psathyrellaceae*, *Spizellomycetales*, and *Aspergillaceae* correlated with SOM, sucrase, nitrate nitrogen, and available phosphorus, reflecting their preference for nutrient-rich environments, while *Ascodesmidaceae* showed dependence mainly on sucrase and phosphorus resources.

Overall, these findings demonstrate that soil physicochemical conditions and enzymatic activities are key determinants of microbial community structure in saline–alkali soils. Specifically, pH, salinity, SOM, and nitrogen and phosphorus availability are the primary drivers of bacterial community composition (Liu et al., 2020; Gong et al., 2021). Enhanced enzyme activities—particularly alkaline phosphatase, sucrase, and polyphenol oxidase—facilitate carbohydrate and organic matter degradation, improving microbial nutrient availability and soil remediation efficiency (Xie et al., 2017; Jyoti et al., 2018; Wong Min et al., 2024). Therefore, regulating SOM, nutrient supply, and salinity, together with optimizing enzyme activities, not only promotes microbial functionality but also contributes to soil quality improvement and the sustainable utilization of saline–alkali land. Future studies should integrate molecular ecology and network analysis to further elucidate the mechanistic linkages between soil properties and microbial ecological functions, providing theoretical support for maintaining soil health and enhancing ecosystem services in saline–alkali regions.

## 5 Conclusion

This study demonstrated that the combined application of maize straw incorporation and irrigation improvement effectively enhanced the remediation of saline–alkali soil by synergistically regulating soil physicochemical properties and microbial community dynamics. The improvement measures significantly increased soil organic matter and available nutrients while reducing salinity and electrical conductivity, thereby optimizing the water–salt balance and nutrient environment. Enhanced sucrase activity and reduced polyphenol oxidase activity reflected improved organic carbon utilization and more efficient nutrient cycling. Microbial community analysis revealed an adaptive reorganization characterized by decreased halotolerant taxa (e.g., *Flavobacteriaceae*, *Rhodobacteraceae*) and enrichment of carbon- and nitrogen-cycling groups (e.g., *Sphingomonadaceae*, *Cyanobiaceae*, *Ascodesmidaceae*). Functional prediction further indicated that genes related to carbohydrate, lipid, and amino acid metabolism were significantly upregulated, suggesting increased microbial energy production and metabolic versatility. Overall, these findings indicate that maize straw incorporation combined with irrigation improvement not only enhances soil fertility and ecological stability but also promotes a functional restructuring of soil microbial communities, providing a robust scientific and practical basis for the sustainable management of saline–alkali land. Future research should focus on evaluating the long-term effects of straw incorporation under continuous management, comparing different straw incorporation rates and modes, and assessing their interactions with irrigation regimes across soil depths and regions. Such studies will be essential for optimizing improvement strategies and ensuring their long-term ecological and agronomic sustainability.

## Data Availability

The datasets presented in this study can be found in online repositories. The names of the repository/repositories and accession number(s) can be found in this article/Supplementary material.
