# Supplementary material for: Interactive effects of maize straw incorporation and improved irrigation on soil physicochemical properties and microbial community structure in saline–alkaline soil
Source: Front Microbiol. 2026 Feb 4;17:1752596. doi: 10.3389/fmicb.2026.1752596 (PMC12913376; doi:10.3389/fmicb.2026.1752596)
Supplement: Supplementary file 1 [file Table_1.docx]

**Supplementary**

**Table S1: Key Soil Physicochemical Properties and Enzyme Activities**

| Index | Soil Layer | Before Improvement (YCK) (Mean ± SD) | After Improvement (YD) (Mean ± SD) | Change Rate (%) | p-value |
| --- | --- | --- | --- | --- | --- |
| Organic Matter (g·kg⁻¹) | 0–10 cm | 6.96 ± 0.48 | 13.00 ± 3.78 | +86.73 | < 0.05 |
|  | 10–20 cm | 6.96 ± 0.48 | 8.00 ± 0.56 | +14.87 | > 0.05 |
| Ammonium Nitrogen (mg·kg⁻¹) | 0–10 cm | 28.82 ± 1.37 | 34.98 ± 6.05 | +21.38 | > 0.05 |
|  | 10–20 cm | 28.82 ± 1.37 | 32.00 ± 4.20 | +11.03 | > 0.05 |
| Nitrate Nitrogen (mg·kg⁻¹) | 0–10 cm | 264.00 ± 16.00 | 304.00 ± 55.43 | +15.15 | > 0.05 |
|  | 10–20 cm | 264.00 ± 16.00 | 285.00 ± 32.10 | +7.95 | > 0.05 |
| Salinity (mg·g⁻¹) | 0–10 cm | 7.59 | 3.52 | -53.56 | < 0.05 |
|  | 10–20 cm | 7.34 | 4.44 | -39.50 | < 0.05 |
| Polyphenol Oxidase (nmol·h⁻¹·g⁻¹) | 0–10 cm | 1841.64 ± 289.38 | 1301.04 ± 179.49 | -29.35 | < 0.05 |
|  | 10–20 cm | 1838.04 ± 290.17 | 1322.67 ± 305.63 | -28.04 | < 0.05 |
| Alkaline Phosphatase (mg·d⁻¹·g⁻¹) | 10–20 cm | 127.91 ± 46.54 | 33.52 ± 21.36 | -73.80 | < 0.05 |
| Sucrase (mg·d⁻¹·g⁻¹) | 0–10 cm | 2.49 ± 0.77 | 5.21 ± 0.96 | +109.23 | < 0.05 |
